# Elastic band resistance training increases adropin and ameliorates some cardiometabolic risk factors in elderly women: A quasi-experimental study

**DOI:** 10.1186/s13102-022-00571-6

**Published:** 2022-10-07

**Authors:** Akbar Azamian Jazi, Esmaeil Moradi Sarteshnizi, Mahshid Fathi, Zahra Azamian Jazi

**Affiliations:** 1 Department of Sport Sciences, Shahrekord University, Shahrekord, Iran; 2 Department of Cardiology, Faculty of Medicine, Isfahan University of Medical Sciences, Isfahan, Iran

**Keywords:** Elderly, Resistance training, Adropin, Cardiometabolic risk factors

## Abstract

**Background:**

The decline in adropin over the aging process is associated with cardiometabolic risks, and resistance training may affect this decline. We hypothesized that elastic band resistance training (EBRT) would be an effective exercise for increasing adropin and improving the cardiometabolic profile in elderly women.

**Methods:**

We randomly assigned 28 overweight elderly women (age = 74.04 ± 4.69 years) into one of two groups, EBRT (n = 14) or control (CON; n = 14), to participate in a 12-week (3d/wk) supervised EBRT program. The serum levels of adropin and cardiometabolic risk factors were evaluated at baseline and after the intervention. The exercise training protocol consisted of three supervised training sessions (55 min) per week for 12 weeks. Data were analyzed using two-way repeated-measures ANOVA and Pearson correlation coefficient.

**Results:**

EBRT significantly increased serum adropin levels (*p* = 0.026), number of repetitions in the 30-second chair-stand test (*p* = 0.016), and number of repetitions in the 30-second arm curl test (*p* = 0.032). Moreover, EBRT significantly decreased serum levels of insulin (*p* = 0.035), TNF-α (*p* = 0.046), hsCRP (*p* = 0.037), and insulin resistance (*p* = 0.045) as well as body fat percentage (*p* = 0.023). There were no significant between-group differences (*p* > 0.05) in glucose, TC, TG, LDL-C, HDL-C, BMI, and WHR; however, glucose, TC, TG, and BMI significantly changed in the EBRT group (within-group). Furthermore, adropin correlated with body fat percentage (p = 0.020) and BMI (p = 0.014) at pretest and with body fat percentage at posttest (p = 0.016), however, delta values were not significantly related. No correlation was observed between adropin and other biomarkers at any stage of the study.

**Conclusion:**

EBRT can increase serum adropin levels, which has been associated with improved insulin sensitivity, inflammation, body fat percentage, and physical function in overweight elderly women.

## Background

Aging involves a set of physiological alterations that lead to several complications such as hormonal imbalance, mitochondrial dysfunction, cellular senescence, and chronic degeneration [1–3]. Aging is a major risk factor for lipid profile disturbances [4], endocrine dysfunction [5], and cardiovascular morbidity in healthy women [6]. Cardiometabolic morbidity and mortality in older adults (≥ 60 years of age) are higher than in younger adults [7]. Adropin is a peptide hormone, which affects cardiometabolic control, and aging can affect its circulating levels [8–12]. It seems that exercise training can positively influence the adropin levels and cardiometabolic risk factors in sedentary adults [8, 9, 11].

Blood adropin levels are inversely associated with age [9, 11, 12], where individuals aged more than 40 years have shown lower plasma adropin levels than those aged less than 30 years [11, 12]. It is well known that estrogen levels decline rapidly in the menopause stage. It has been recently reported that hepatic adropin gene expression is regulated by estrogen, and its downregulation is associated with adverse metabolic phenotypes in ovariectomized mice [13, 14]. Adropin can be linked with inflammation, so a decrease in adropin levels may be associated with an increase in inflammation [15]. A number of clinical studies have demonstrated decreased expression of adropin in various inflammatory diseases, its potential anti-inflammatory effects, and its negative correlation with inflammatory cytokine levels. By decreasing fat accumulation, adropin may decrease macrophage infiltration, thereby improving inflammation. It exerts anti-inflammatory effects on atherosclerosis through the regulation of the macrophage phenotype [16]. Also, the reduced levels of adropin are accompanied by dyslipidemia, high body mass index (BMI), and cardiovascular disease [9, 17]. Adropin has a significant negative correlation with the levels of cytokines and hypersensitive C-reactive protein (hsCRP) [16]. It also reduces the mRNA expression of pro-inflammatory cytokines, such as TNF-α through inducible NOS expression in the pancreas and liver [18]. Adropin is associated with mild suppression of the endogenous rate of glucose production, and its deficiency has a negative effect on glucose homeostasis [17]. Also, low adropin levels are associated with insulin resistance (IR) [9, 17, 19]. It plays an important role in metabolic regulation and can lead to lower insulin sensitivity [15, 20]. In terms of physical activity, an increasingly sedentary lifestyle in older people is associated with decreased adropin levels [17, 21, 22].

Regular physical exercise is an effective non-pharmacological preventive and therapeutic intervention to reduce the risks of various conditions associated with advanced age. Progressive resistance training can improve cardiometabolic health in older adults [23]. Given that older individuals may not have proper access to free weights or gym equipment [24], elastic band resistance training (EBRT) can be a great alternative to this equipment [25]. EBRT is an effective method for improving muscle strength in the elderly [26]. Elastic bands are more accessible, portable, and more affordable than free weights and resistance training devices, and their use is increasing day by day [27]. Muscle activation in EBRT and other resistance training with free weights is almost identical [28]. Elastic band exercise can improve the functional capabilities of patients [29]. Colado et al. reported that EBRT has positive effects on body strength and body composition [30]. Furthermore, strength training and EBRT had a significant effect on the lipid profile in postmenopausal women [31]. Therefore, in this study, EBRT was used as an exercise intervention.

In summary, low adropin levels are associated with an unfavorable cardiometabolic profile and can have devastating consequences for older people, and it seems that exercise training may have beneficial effects on this process. Therefore, this study hypothesized that EBRT would positively change adropin levels and also improve the cardiometabolic profile.

## Methods

### Study participants and design

**Fig. 1:**
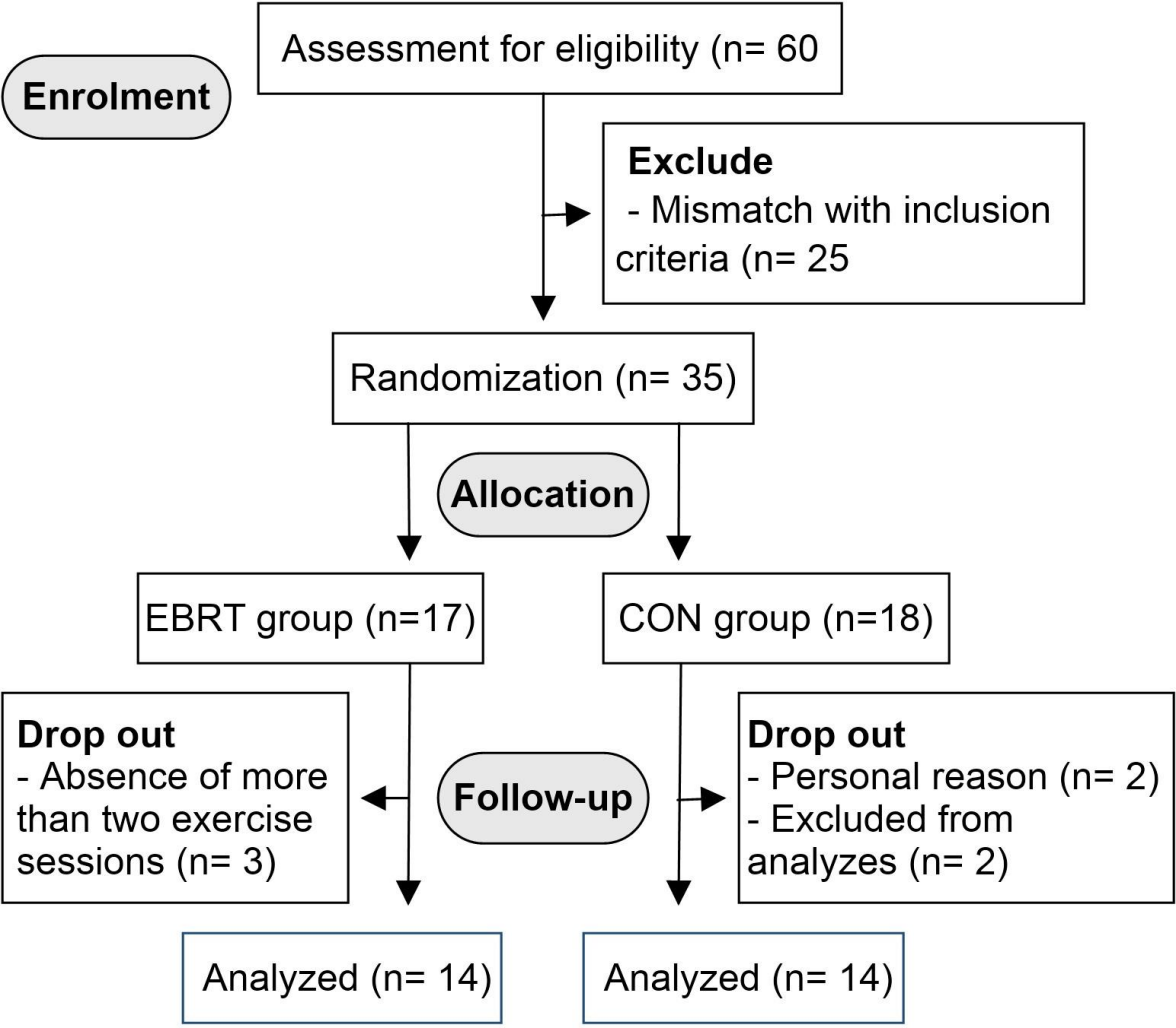
CONSORT flow diagram

Sixty healthy overweight older women (age = 74.04 ± 4.69 years) who are living in the elderly nursing home were screened for the study (Fig. 1). The main inclusion criteria were as follows: (1) overweight elderly women aged 65–80 years, (2) physically independent, (3) no musculoskeletal, cardiovascular, neurological, mental, or any chronic diseases that could inhibit exercise performance or put participants at risk from exercising, (4) no regular exercise training for at least six months before participating in this study, and (5) medical clearance for participation. Those participants who could not complete the training program or had medical conditions that made it unsafe for them to participate in the exercise program were excluded from the study.

The sample size was estimated by G*Power 3.1.9.2 software. The minimum sample size was calculated to be 24, considering the previous studies [22, 28, 32], and based on an effect size of 0.30, an α-level of 0.05, and a power of 0.80. Finally, considering possible dropouts due to the COVID-19, 35 participants were recruited. Then, they were randomly divided into a CON group (n = 18) and an EBRT group (n = 17). The allocation was concealed and stratified for homeostasis model assessment (HOMA-IR) using random block allocation (2 blocks). The HOMA-IR was calculated as (fasting glucose [mg/dl] × fasting insulin [µU/mL])/405 [33]. The control group was instructed to maintain their usual physical activity during the study. Assessments occurred at baseline approximately one week before starting the intervention, and 48 h after 12 weeks of intervention. All assessments were blinded to the allocation of the participants.

### Elastic bands resistance training intervention

The participants in the experimental group were instructed on how to use elastic bands (TheraBand, The Hygenic Corporation, Akron, OH, USA) before starting the EBRT intervention. The EBRT protocol was designed based on a previous study and the guidelines of the American College of Sports Medicine [28, 34]. Table 1 outlines the exercise protocol. EBRT was performed three times a week (on non-consecutive days) for 12 weeks in small groups of participants (n ≤ 6) and under the supervision of two qualified exercise scientists. The correct exercise technique, rest intervals, and compliance with the exercise program were closely monitored. Each session lasted 55 min and consisted of 10 min of warm-up followed by 40 min of EBRT and finally five minutes of cooling down. The EBRT was conducted for the major muscle groups, including arms, shoulder, abdomen, back, and legs. One to three sets of 8–12 repetitions during the first 3 weeks and then three sets of 8–12 repetitions during the rest of the study period, were performed by the participants through the full range of motion for each exercise, and the training volume and intensity progressively increased [34]. In this study, the resistance levels of elastic bands begin and increase from the lowest resistance (yellow) to the highest (black) progressively. The Borg scale was used to estimate the exercise intensity and individualize the intervention for each participant. Moreover, exercise intensity was controlled by this scale at the point of 12–14. When the perceived effort was rated as about 13 points (somewhat hard), the resistance exercise intensity was progressively increased using the next elastic band color. Moreover, when the participants could not tolerate the resistance intensity of the next band color, the previous one was maintained for an additional session [28, 34].

**Table 1:** Exercise protocol

	TheraBand colors					Exercise loading		
Weeks	Yellow	Red	Green	Blue	Black	Repetitions	Sets	RPE^a^
1	X					8–10	1–2	12–14
2	X					8–10	2–3	12–14
3	X					10–12	2–3	12–14
4	X					10–12	3	12–14
5		X				8–10	3	12–14
6		X				10–12	3	12–14
7			X			8–10	3	12–14
8			X			10–12	3	12–14
9				X		8–10	3	12–14
10				X		10–12	3	12–14
11					X	8–10	3	12–14
12					X	10–12	3	12–14

X = the used TheraBand color

^a^ Ratings of perceived exertion according to the Borg scale

### Lower and upper limb strength tests

These tests were used to evaluate the effectiveness of 12-weeks of EBRT on the lower and upper limb strength. The 30-second chair-stand test using a chair without arms (seat height: 43.2 cm) was used to assess lower-body strength. The score of each subject was the total number of the full stand from a seated position within the 30-s period [35]. The 30-second arm curl test was used to assess upper-body strength, in which the highest number of full elbow flexion to extension in 30 s holding a hand weight of 2.3 kg was accounted as the score [35].

### Anthropometry and body composition

Standing height was measured with the participants’ shoes off. Weight, BMI, waist-to-hip ratio (WHR), and body fat percentage (three-site skinfold method: triceps, suprailiac, thigh) were assessed before and after the EBRT program at the same time of the day.

### Blood sample collection and analysis

Fasting blood samples were obtained from the cubital vein using standard procedures at 8–9 am; the first time was one week before the 12-week training period, and the second time was 48 h after the last session of the 12-week training intervention. The samples were centrifuged at 3000 rpm for 20 min, and the serum was stored at -70 ° C for future analysis.

Serum adropin was measured in duplicates using a commercially available ELISA kit (Catalog No: CK-E90267, Eastbiopharm Co. Ltd, Hangzhou, China) according to the manufacturer’s instructions (Assay range: 5 ng/L – 1000 ng/L, Sensitivity: 2.49 ng/L). Insulin, glucose, total cholesterol (TC), high-density lipoprotein cholesterol (HDL-C), low-density lipoprotein cholesterol (LDL-C), triglycerides (TG), and hsCRP were assessed using standard techniques.

### Statistical analysis

Statistical analysis was performed using SPSS (SPSS Inc., Chicago, IL, USA). The Shapiro–Wilk test was used to check the normality. Comparisons of baseline variables were made using the independent student t-test. Group comparisons were performed using two-way repeated measures ANOVA [group (CON group and EBRT group) × time (before and after 12 weeks)]. Additionally, the Bonferroni test was used for pairwise comparisons. Furthermore, the relationships between serum adropin and other variables were evaluated with the Pearson product-moment correlation analysis. A *p* value < 0.05 was considered statistically significant. The graphs were plotted using GraphPad Prism (version 8.3.0 for Windows; Graphpad Software, San Diego, CA).

## Results

Of the 60 volunteers screened for the study, 35 were allocated to the EBRT group or CON group. Seventeen participants commenced the EBRT, with 14 of them completing the program. Eighteen participants in the CON group commenced the study, with 14 completions (Fig. 1). Therefore, the data were analyzed in 28 participants, consisting of 14 participants in the CON group and 14 participants in the EBRT group. Participant characteristics are shown in Table 2. There were no significant differences in any baseline characteristic (*p* > 0.05) between the groups.

**Table 2:** Participant baseline characteristics

Variables	EBRT group (n = 14)	CON group (n = 14)	T	P
Age (years)	73.29 ± 5.44	74.79 ± 3.87	-0.841	0.408
Height (cm)	151.28 ± 4.79	150.36 ± 4.45	0.531	0.600
Weight (kg)	63.71 ± 8.76	64.86 ± 9.63	-0.328	0.745
BMI (kg/m^2^)	27.82 ± 3.46	28.59 ± 3.32	-0.603	0.552
WHR	0.96 ± 0.08	0.97 ± 0.07	-0.466	0.645
Body fat percentage	36.05 ± 1.96	36.36 ± 2.49	-0.366	0.717
HOMA-IR	0.87 ± 0.15	0.92 ± 0.23	-0.734	0.470
Insulin (mg/dl)	4.01 ± 0.54	4.28 ± 0.84	-1.002	0.325
Glucose (mg/dl)	97.21 ± 7.44	96.50 ± 8.51	0.236	0.815
TG (mg/dl)	146.57 ± 39.13	125.21 ± 30.93	1.602	0.121
TC (mg/dl)	185.36 ± 27.61	176.50 ± 27.38	0.852	0.402
LDL-C (mg/dl)	123.21 ± 18.75	122.86 ± 18.57	0.051	0.960
HDL-C (mg/dl)	41.36 ± 5.00	42.64 ± 4.16	-0.739	0.466
30-second chair stand test (reps)	10.43 ± 1.91	11.29 ± 1.44	-1.342	0.191
30-second arm curl test (reps)	16.14 ± 2.44	16.71 ± 2.61	-0.597	0.555
Adropin (ng/L)	100.73 ± 18.56	106.33 ± 22.50	-0.719	0.479
TNF-α (pg/ml)	5.79 ± 0.87	5.84 ± 0.86	-0.155	0.878
hsCRP (ng/ml)	1714.86 ± 508.33	1895.79 ± 727.69	-0.763	0.453

Values are presented as mean ± SD

BMI: Body mass index; WHR: waist-to-hip ratio

### Changes in serum adropin levels

**Fig. 2:**
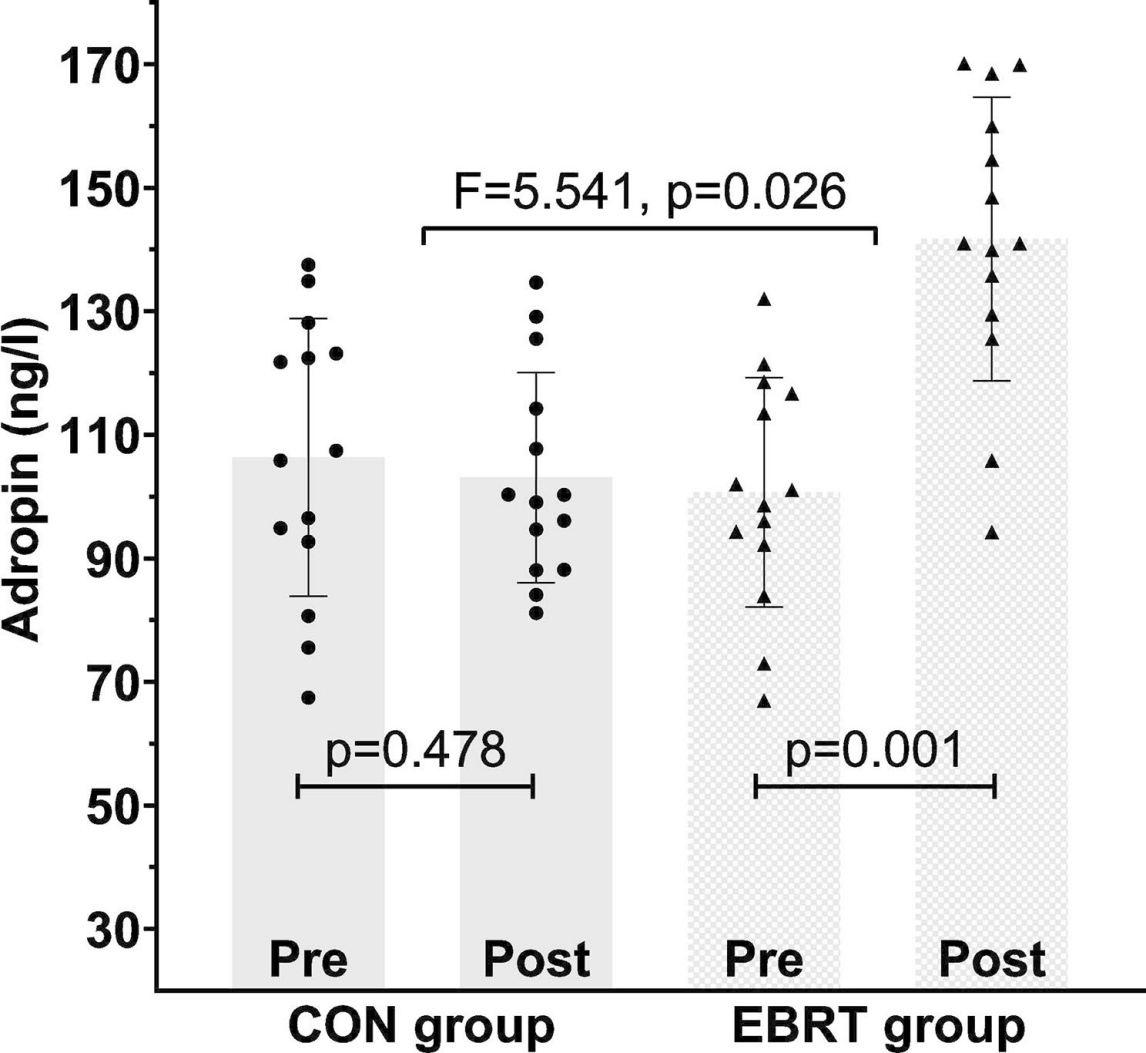
Changes in adropin levels following 12 weeks of EBRT

The difference between EBRT and CON groups was significant for adropin levels after 12 weeks of exercise intervention (F (1, 26) = 5.541, *p* = 0.026, partial eta squared = 0.176). The EBRT group showed an increase of 40.71% (*p* = 0.001, from 100.73 ± 18.56 to 141.74 ± 22.97 ng/L), whereas, no significant changes were found in the CON group (*p* = 0.478, from 106.33 ± 22.50 to 103.07 ± 17.02 ng/L) (Fig. 2).

### Changes in serum levels of TNF-α and hsCRP

**Fig. 3:**
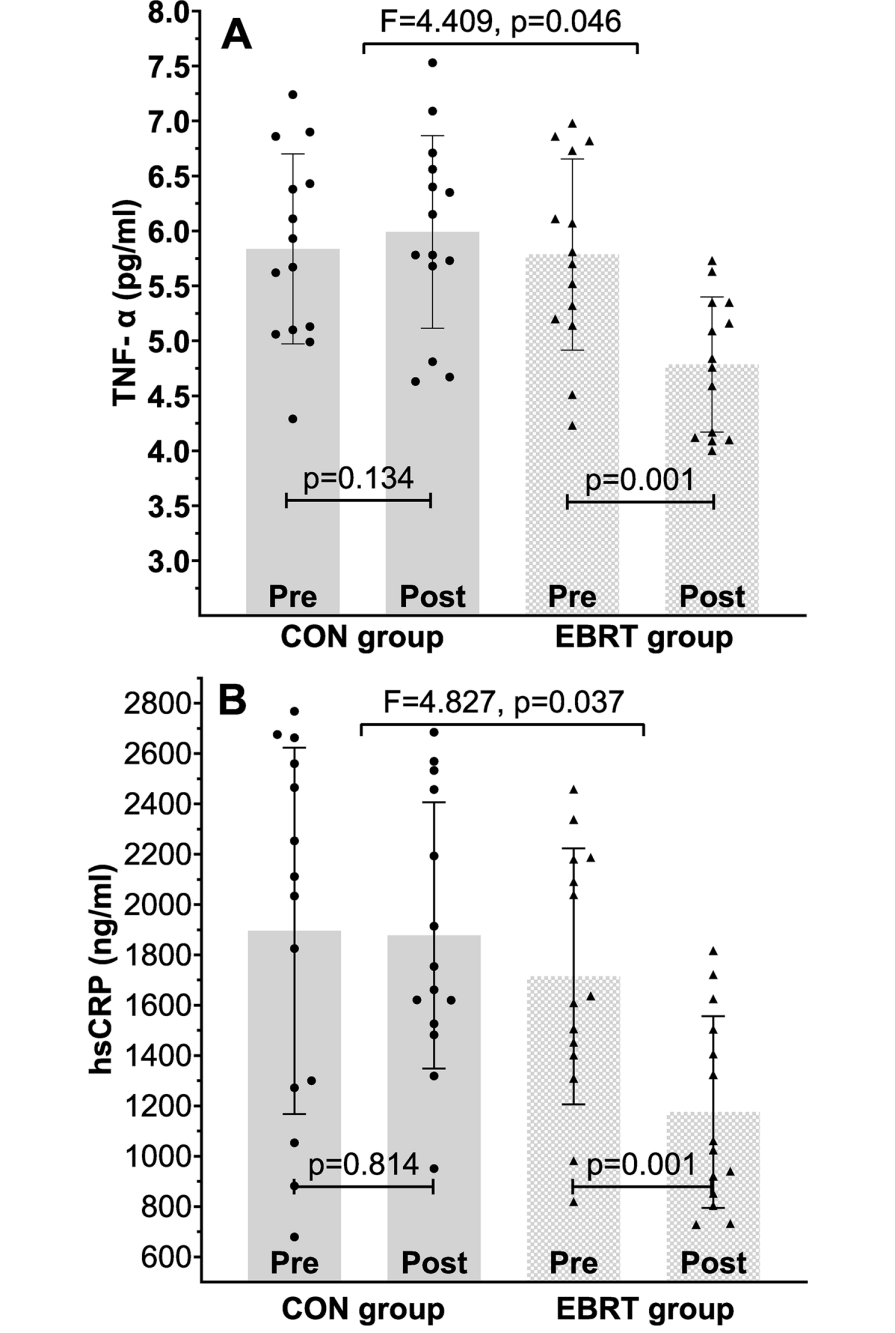
Changes in TNF-α (**A**) and hsCRP (**B**) following 12 weeks of EBRT

There was a statistically significant reduction in TNF-α (F (1, 26) = 4.409, *p* = 0.046, partial eta squared = 0.145) and hsCRP (F (1, 26) = 4.827, *p* = 0.037, partial eta squared = 0.157) levels in the EBRT group compared with the CON group (Fig. 3).

### Changes in insulin resistance, insulin, and glucose

**Fig. 4:**
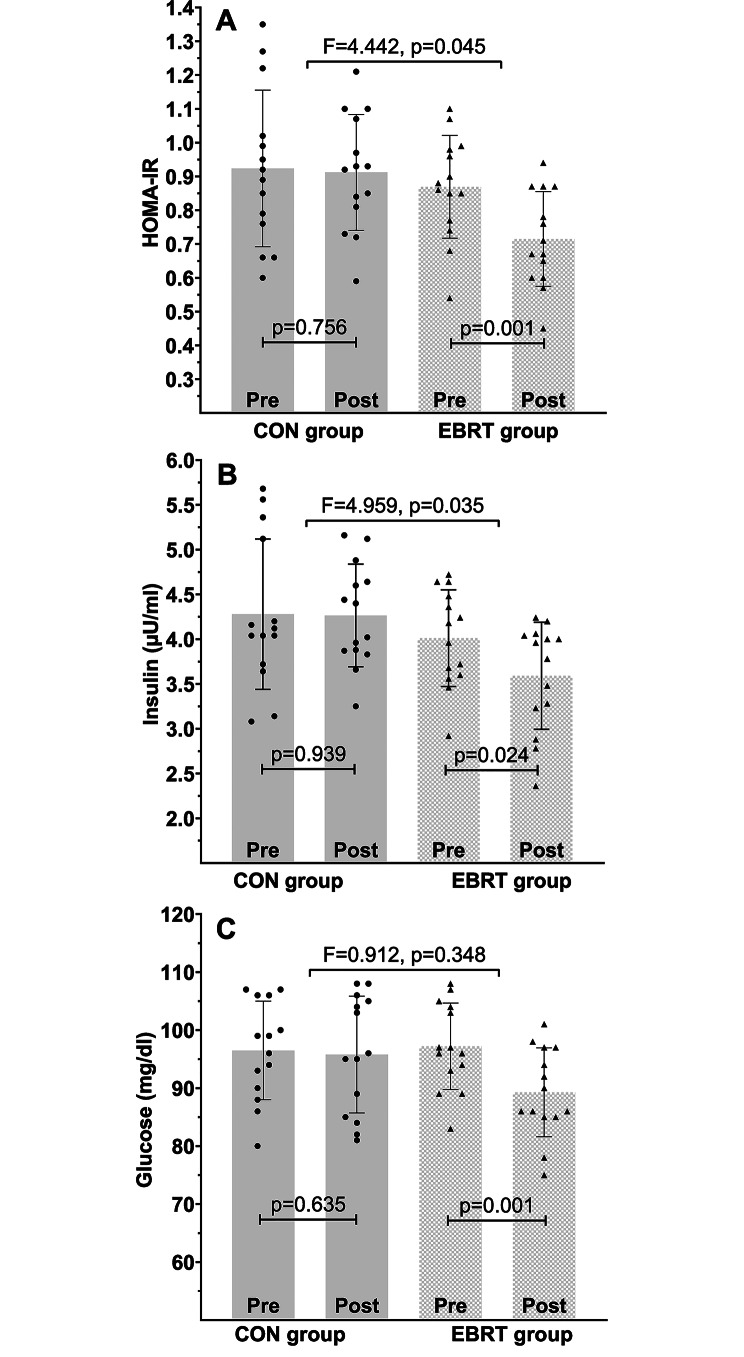
Changes in HOMA-IR (**A**), insulin (**B**), and glucose (**C**) following 12 weeks of EBRT

The results on HOMA-IR, insulin, and glucose are presented in Fig. 4. HOMA-IR significantly improved with ERBT compared to the control condition (F (1, 26) = 4.442, *p* = 0.045, partial eta squared = 0.146). Insulin levels in the ERBT group significantly decreased compared to the CON group (F (1, 26) = 4.959, *p* = 0.035, partial eta squared = 0.160). There were no significant between-group differences in glucose levels after 12 weeks of EBRT (F (1, 26) = 0.912, *p* = 0.348, partial eta squared = 0.034), despite the significant decrease in its levels in the EBRT group.

### Changes in BMI, WHR, body fat percentage, and lipid profile

The results on BMI, WHR, and body fat percentage can be seen in Table 3. There were no significant differences between the EBRT and the CON groups in terms of BMI (F (1, 26) = 0.876, *p* = 0.358) and WHR (F (1, 26) = 0.222, *p* = 0.641) after 12 weeks of exercise intervention. The body fat percentage significantly reduced in the EBRT group compared with the CON group (F (1, 26) = 5.868, *p* = 0.023). As shown in Table 3, there were no significant between-group differences over time for any of the lipid profile components (*p* > 0.05).

**Table 3:** The effects of 12 weeks of EBRT or control period on BMI, WHR, body fat percentage, and lipid profile parameters

	EBRT group			CON group			Partial Eta Squared
	Mean Differences ± Std. Error	95% Confidence Interval		Mean Differences ± Std. Error	95% Confidence Interval		
		Lower Bound	Upper Bound		Lower Bound	Upper Bound	
BMI (kg/m2)	0.577 ± 0.136^*^	0.296	0.857	-0.284 ± 0.136^*^	-0.565	-0.004	0.033
WHR	0.005 ± 0.006	-0.007	0.018	0.006 ± 0.006	-0.006	0.019	0.008
Body fat percentage^#^	3.642 ± 0.254^*^	3.120	4.165	0.150 ± 0.254	-0.737	0.762	0.184
TG (mg/dl)	17.429 ± 6.122^*^	4.845	30.012	-6.643 ± 6.122	-19.226	5.940	0.018
TC (mg/dl)	17.929 ± 4.342^*^	9.003	26.855	-10.286 ± 4.342^*^	-19.212	-1.360	0.012
LDL-C (mg/dl)	1.714 ± 1.105	-0.557	3.986	-1.143 ± 1.105	-3.415	1.129	0.001
HDL-C (mg/dl)	-1.000 ± 0.494	-2.016	0.016	-0.286 ± 0.494	-1.301	0.730	0.012

^#^Significant difference between EBRT and CON groups (P < 0.05). *Within-group significant differences (P < 0.05) (Pre: before training intervention; Post: after training intervention)

### Changes in physical function

**Fig. 5:**
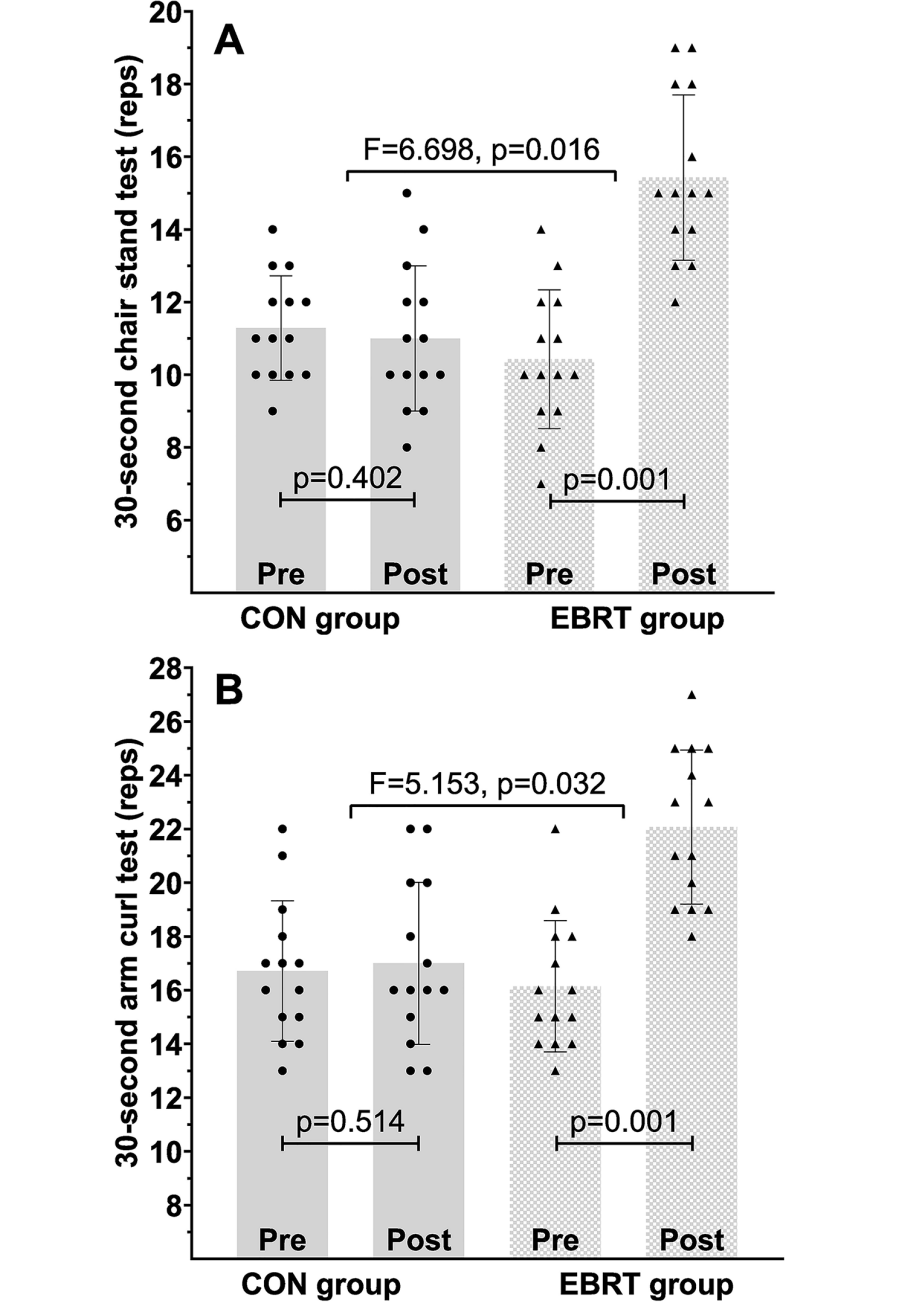
Changes in physical function following 12 weeks of EBRT. **A** 30-second chair-stand test. **B** 30-second arm curl test

The 30-second chair-stand test results significantly improved in the EBRT group compared with the baseline (*p* = 0.001) and the CON group (F (1, 26) = 6.698, *p* = 0.016, partial eta squared = 0.205) (Fig. 5 A). The 30-second arm curl test results significantly improved in the EBRT group compared with the baseline (*p* = 0.024) and the CON group (F (1, 26) = 5.153, *p* = 0.032, partial eta squared = 0.165) (Fig. 5B).

### Correlations between adropin and other biomarkers

Correlation analysis revealed that adropin negatively correlated with body fat percentage at both pretest (*r* = 0.439, *p* = 0.020) and posttest (*r* = 0.631, *p* = 0.016), however, delta values between the two measures were not significantly related. Moreover, adropin only correlated with BMI at pretest (*r* = 0.459, p = 0.014). No correlation was observed between adropin and other biomarkers at any stage of the study.

## Discussion

In this study, serum adropin levels and cardiometabolic risk factors were analyzed to evaluate whether 12 weeks of EBRT improved aging-related changes in these variables in overweight elderly women.

The main findings of this study are that EBRT significantly increased serum adropin levels, improved insulin sensitivity, and improved physical function. Moreover, TNF-α, hsCRP, insulin levels, and body fat percentage significantly decreased following EBRT.

Adropin is expressed in the brain, heart, liver, kidney, skeletal muscle, pancreas, vascular endothelial cells, and small intestine [20, 36], and it is of great importance in maintaining metabolic homeostasis; therefore, a decrease in its concentration can increase IR in older men and women [17]. It is indicated that adropin, as an endocrine factor, can reduce IR, and can play an important role in the regulation of energy metabolism [19]. Moreover, reduced levels of adropin are accompanied by cardiovascular disease and dyslipidemia [9]. The results of this study indicated that 12 weeks of EBRT increased serum adropin levels. This result is consistent with a study by Rezaeinezhad et al. (2022), who found that serum adropin levels significantly increased after six weeks of circuit resistance training in dormitory young male students [37]. Moreover, Fujie et al. have demonstrated that serum adropin levels increased after eight weeks of aerobic exercise training with an intensity of 60 to 70% of VO_2peak_ in 40 healthy middle-aged and older subjects [22]. Therefore, considering these studies and our results, it seems that regardless of the effect of aging, both resistance and aerobic training could increase adropin levels.

In this study, the body fat percentage decreased after EBRT. As people age, the body fat mass will gradually increase compared to lean body mass. Adipose tissue is a “crossroads” of energy homeostasis, inflammation, and atherosclerosis [38]. Macrophages in the adipose tissue are the source of inflammatory cytokines, such as TNF-α, that play a significant role in the inflammatory process [39, 40]. It has been reported that a decrease in adropin levels is associated with an increase in body fat mass [17]. Moreover, this obesity-dependent decrease in adropin levels may lead to more severe IR and dyslipidemia [17].

Adropin has a potential anti-inflammatory effect and a negative correlation with the expression levels of inflammatory cytokines [16]. However, further research is needed to confirm whether adropin can alter the phenotype of macrophages; it may regulate the pro-inflammatory or anti-inflammatory phenotypes of macrophages through up-regulating the expression of peroxisome proliferator-activated receptor gamma (PPAR-γ) [18]. In other words, PPAR- may be a key target for adropin’s anti-inflammatory properties [16]. It has also been shown that PPAR-γ plays an important role in inflammation and metabolism in macrophages [41]. Furthermore, PPAR-γ activation can reduce macrophage infiltration and adipose tissue inflammation. Excessive free fatty acids in metabolic tissues, such as skeletal muscle, pancreas, and liver, can activate inflammatory pathways; on the other hand, adropin can help minimize macrophage infiltration by reducing fat accumulation and consequently can reduce inflammation [16]. Inflammation and insulin resistance can trigger metabolic disorders that activate inflammatory transcription factor nuclear factor-κB (NF-κB) and the inflammatory signaling system, as well as elevated cytokine levels, thus accelerating damage to endothelial cell function and formation of atherosclerotic plaques. Adropin can also exert its possible anti-inflammatory effects through energy metabolism regulation [16].

In this study, an increase in serum adropin levels was associated with improved physical function. Generally, muscle mass, physical strength, and physical function decrease with age. A former study has shown that a reduction in adropin levels in mice is associated with a mild decrease in physical activity [17], and another study also reported a significant increase in physical activity in transgenic female mice that had an increased expression of adropin levels [21]. Adropin as a membrane-bound protein interacts with the brain-specific Notch1 ligand NB3. It may regulate physical activity via the NB-3/Notch signaling pathway [20]. Therefore, the improvement in physical function in this study seems to be associated with elevated adropin levels. Additionally, physical function improvement confirms that the volume and intensity of EBRT have been appropriate.

Although EBRT has a positive significant influence on lipid profile (only TC and LDL-C) after one year of a training program in postmenopausal women [31], the lipid profile components did not significantly change after the exercise intervention in this study, which can be attributed to the shorter duration of the training program in this study.

In this study, adropin only correlated with body fat percentage and BMI at pretest and body fat percentage at posttest, and no correlation was observed between adropin and other parameters at any stage of the study. However, several studies have shown that serum adropin is correlated with some inflammatory markers and metabolic parameters [9, 16, 17, 19]. It is necessary to remember that participants in this study were elderly women, and adropin levels may be affected by the aging process.

There were some limitations in this study. First, all the participants in this study were females, so the results are not generalizable to all older people. Second, although we requested the participants to maintain their usual level of physical activity, it would have been better if we had monitored and controlled it. Third, although we calculated the sample size before enrolling participants in this study, future studies with a larger sample size may be necessary to give more conclusive results. Fourth, although the body fat percentage was measured using the skinfold method, it would have been better if we had measured it by dual-energy X-ray absorptiometry.

We think that the serum levels of glucose, TC, and TG could be affected by more extended duration intervention or more intense exercise training, so we suggest that future studies should be conducted to evaluate the effect of EBRT on these variables. To the best of our knowledge, this is the first study to evaluate the effect of 12 weeks of EBRT on adropin and cardiometabolic risk factors in overweight elderly women, so more comprehensive clinical studies could provide more details. No adverse effects were observed in this study.

## Conclusion

Increased serum adropin levels following 12 weeks of EBRT in overweight elderly women are associated with changes in some of the cardiometabolic risk factors including, a decrease in insulin levels and body fat percentage, and an improvement in insulin sensitivity. In addition, physical function improvement is associated with an increase in adropin levels, which confirms that the volume and intensity of EBRT were appropriate. In summary, it appears that the improvement of some age-related cardiometabolic risk factors is associated with an increase in adropin levels after 12 weeks of EBRT.

## Data Availability

The datasets used and/or analyzed during the current study are available from the corresponding author on reasonable request.

## Abbreviations

EBRT: Elastic band resistance training.
CON: Control.
BMI: Body mass index.
WHR: Waist-to-hip ratio.
IR: Insulin resistance.
hsCRP: Hypersensitive C-reactive protein.
TNF-α: Tumour necrosis factor-alpha.
HOMA-IR: Homeostasis model assessment.
TC: Total cholesterol.
HDL-C: High-density lipoprotein cholesterol.
LDL-C: Low-density lipoprotein cholesterol.
TG: Triglycerides.
PPAR-γ: Peroxisome proliferator-activated receptor gamma.
